# Evaluation of HIV and AIDS knowledge in rural Cameroon men with the use of a questionnaire

**DOI:** 10.11604/pamj.2013.16.141.2964

**Published:** 2013-12-18

**Authors:** Hendt Paul Versteegh, Affuenti Bakia, Hendrik Maria Koopman, Vivian Kraaij, Florens Gerard Adriaan Versteegh

**Affiliations:** 1 Apostolic Hospital, Banga Bakundu, Cameroon; 2 Present: medical student, Erasmus Medical Center, Rotterdam, the Netherlands; 3 Leiden University, Department of Clinical, Health and Neuropsychology, Leiden, the Netherlands; 4 Groene Hart Ziekenhuis, Dept. of Pediatrics, Gouda, the Netherlands

**Keywords:** HIV/AIDS knowledge, Cameroon, men, age

## Abstract

**Introduction:**

HIV/AIDS, the most important health problem in Africa, is the leading cause of death on the continent. Ignorance on HIV/AIDS status will hamper treatment and prevention. To investigate the level of HIV/AIDS knowledge among men in a rural area, we performed a questionnaire study on HIV/AIDS knowledge in men living in Banga Bakundu, a rural village in Cameroon.

**Methods:**

Forty-eight men, aged 17-66 years, were interviewed. They were divided in 2 groups: ≤29 years, being those young enough to be able to have knowledge about HIV/AIDS at the time of their first sexual contact, and those > 29 years who weren't. A semi-structured clinical interview was performed to obtain information about socio-demographic characteristics, sexual activity, knowledge about HIV/AIDS and its prevention.

**Results:**

There is an overall good HIV/AIDS knowledge and what should be done about it. Men with a higher level of education and more HIV/AIDS knowledge seem to take less preventive measures. The differentiation per age group showed that age influenced the data on knowledge and behaviour.

**Conclusion:**

Our data are consistent with other studies. Remarkable is the difference in HIV/AIDS knowledge between the 2 age groups, and the relation between HIV/AIDS knowledge and sexual habits and prevention. Sufficient HIV/AIDS knowledge did not lead to significant changes in sexual behaviour. The questionnaire showed to provide sufficient information and was easy to use. Further research should be performed.

## Introduction

HIV/AIDS is the most important health problem in Africa and is the leading cause of death on the continent. In many countries the average life expectancy has fallen into the 30s. Antiretroviral medications - the only hope for slowing disease progression - are not available to most Africans, and HIV testing is not routinely offered in many areas [1]. Although Southern Africa has been the hardest hit, with several countries having adult HIV prevalence greater than 20 percent, HIV has also spread dramatically in Sub-Saharan countries in the western part of the continent and around the Horn of Africa [2].

Cameroon is a low-income country in the west of sub-Saharan Africa. It is often referred to as “Africa in Miniature” because of its complex cultural, social and geographical diversity [3]. Cameroon's population is estimated at about 19,599 million [4], comprising some 250 ethnic groups, making her a “racial crossroads” in Africa. It is the only country in Africa with English and French as its official languages [3]. The national HIV/AIDS policy officially pays great attention to awareness and prevention programs [3, 5]. Despite these efforts the country has the highest infection rate of HIV type 2 (HIV-2) and the most unusual HIV-1 subtypes in the world [6, 7]. It is suspected that the virus was initially transmitted from chimpanzees to humans in Cameroon, where the emergence of the AIDS pandemic occurred [8, 9].

Prevalence rates in Cameroon vary from 5.5% [10] to 12% [5]. Most HIV transmissions occur through sexual intercourse (90%) [3]. The peak age of reported cases in 2004 was 25-49 years for both men (5.7%) and women (8.4%) [11]. The prevalence is higher in urban than in rural residents. Also it is higher in people with most education [11]. It is thought that HIV/AIDS prevalence on the one hand and relations between men and women on the other determine knowledge, opinions and attitudes relating to HIV/AIDS [12]. The prevention of HIV/AIDS is difficult due to competing social problems, myths concerning condom use and HIV transmission, stigma towards persons living with AIDS, gender inequities, and conflict between practitioners of traditional and Western medicine [2, 12]. Ignorance on HIV/AIDS status will hamper treatment and prevention. Therefore it is important to increase health-related knowledge about HIV/AIDS [1, 2]. Both information about HIV/AIDS and confrontation with HIV/AIDS are considered to be important forces to influence peoples attitude toward HIV/AIDS, the latter being the most forceful one [12].

Many studies on HIV/AIDS in Cameroon have been performed, also on knowledge and attitudes in different ways [1, 5, 10, 12–16] but few studies focused on the importance of HIV/AIDS knowledge in men alone [10, 13]. There is a nationwide program on informing the people about HIV/AIDS since 2001 [17] which started with the creation of the National AIDS Control Committee in 2000. This committee focused on voluntary counselling and testing (VCT) and awareness creation. According to the National Aids Control Committee website the first case of HIV was diagnosed in Cameroon in 1986.

To investigate the level of knowledge on HIV/AIDS among men in a rural area we performed a questionnaire study on the knowledge about HIV/AIDS in men living in Banga Bakundu, a rural village, Southwest Province, Cameroon. Since HIV/AIDS was discovered in the early 1980s [18–20] we also looked whether men becoming sexually active before there was general knowledge about HIV/AIDS might have a different level of knowledge and attitude compared to younger men.

## Methods

### Sample

From January until April 2006 a total of 48 men, aged 17-66 years (mean 32, SD 12), were interviewed. The age of their partners (of the 36 who had a partner) ranged 15-48 years (mean 28, SD 9). All men gave oral informed consent. Demographic data are in Table 1. The men originated from 26 different tribes, the most prevalent being Oroco and Metta (each 5), and Menka and Bakundu (each 4). Almost 44% were farmers, a little more than 18% students, the other 38% had all kind of different jobs. About 75% was married or had a stable relation. The median duration of the marriage was approximately 13 years. Twenty men had 1-3 children, 11 had 4 or more children. There were 105 children in total (61 girls). Most men finished primary school, more than 1/3 completed a secondary education. Their partners were equally educated.

**Table 1:** Demographic data

**N**		48	
**Mean age (years)**		32.2	range 17 - 66
**Marital status**			
	Married	24	
	Relationship	12	
	Single	10	
	Divorced	2	
**Education**	No education	1	
	Started primary school	47	
	Finished primary school	44	
	Started secondary school	24	
	Finished secondary school	18	
**Tested on HIV**			
	Yes	19	
	No	29	
**Result HIVtest**			
	Not tested	29	
	Says to be positive	0	
	Says to be negative	16	
	Says he never got the result	3	
**Mean age first sexual contact (n = 47) (years)**		17.9	range 10 - 23
**Used condom during first sexual contact (n = 47)**			
	Yes	14	
	No	33	
**HIV knowledge**			
	No knowledge	10	
	Some knowledge	10	
	Knowledge	20	
	Good knowledge	8	

Because HIV/AIDS was discovered in the early 1980s we divided the men in 2 groups: group I: ≤29 years, being those young enough to be able to have had knowledge about HIV/AIDS at the time of their first sexual contact, and group 2: those > 29 years who were not.

### Procedure

This study was performed by one interviewer (HPV) among randomly selected men older than 16 years of age visiting the outpatient clinic of the Apostolic Hospital, a 75 bed hospital in Banga Bakundu, Southwest Province, a rural English speaking area in Cameroon. Following oral informed consent, a semi-structured clinical interview was performed to obtain information about socio-demographic characteristics, sexual activity, knowledge about HIV/AIDS and its prevention (appendix 1).

### Measures

To measure HIV knowledge we developed a questionnaire (see appendix 1), categorizing given answers in 4 domains: 1: demographic data, 2: sexual habits (4 items), 3: HIV knowledge (6 items), 4: prevention (5 items). The answers were written down (HPV), categorized and put in a database (HMK).

### Statistical Analyses

Statistical analysis was performed using SPSS-pc-17. Concerning data analysis we performed frequencies, descriptive, Pearson correlation coefficients and Student t test.

## Results

At the time of the interview 40% of the men had no or little knowledge on HIV, the other 60% reasonable to good knowledge (Table 1). Around 40% was tested for HIV, of which most reported they were negative (16 out 19, the other 3 told they never received the result).

The age of first sexual contact varied between 10 and 23 years (mean 18, SD 3) and about 30% reported condom use during this first contact. More than 75% of the interviewed men reported that they had no knowledge about HIV at the time of their first sexual contact.

Thirty-four out of 36 (94%) responders reported to have a sexual relation at the time of the interview. Eight out of 34 (24%) reported to have a sexual relation with yet another woman, irrespective to their marital status. Those 8 men reported to abstain from sex as most used preventive measure!

Three-quarter of the men told to use some form of prevention against HIV. The majority of the men (66%) indicated that they would discuss the HIV/AIDS topic with their partner, and would tell their partner when they would test HIV positive, irrespective to age, level of education, or level of knowledge on HIV. More than 55% would stay with their partner when she would tell him she tested HIV positive. Four men would separate. Twenty-six out of 31 (84%) men who had children discussed HIV/AIDS with their children or would do so when they are old enough.

Comparing group I (≤29 years) with group II (>29 years) (Table 2) we found 4 significant differences, namely HIV/AIDS knowledge at 1^st^ sexual contact, the use of a condom at the time of their 1^st^ sexual contact, being a student or not, and being married or not, all in favour of the younger group. Although men ≤ 29 had more knowledge on HIV/AIDS at the time of their 1^st^ sexual contact than those > 29 (t-test 0.01), at the time of the survey the older group had catched up (t-test 0,80, NS).

**Table 2:** Students test (mean and SD) of the 2 age groups

	≤ 29 years			> 29 years				
	N	mean	SD	N	mean	SD	t	Sig 2 tailed
							Equal variance not assumed	
HIV/AIDS knowledge at 1st sexual contact	25	0.40	0.50	22	0.00	0.00	4.00	0.01*
Students vs non-students/those who work	19	1.47	0.51	11	1.00	0.00	4.03	0.01*
Marital state	25	0.92	0.81	20	1.75	0.55	-4.07	0.01*
Education	26	10.08	3.59	22	9.36	4.20	0.63	0.54
Religion	26	2.46	1.30	22	2.73	0.99	-0.80	0.43
Education partner	16	9.50	3.93	20	8.35	3.98	0.87	0.39
HIV/AIDS knowledge	26	1.58	0.99	22	1.50	1.06	0.26	0.80
Tested for HIV/AIDS	26	0.62	0.50	22	0.59	0.50	0.17	0.87
Result HIV	10	19.80	41.74	9	11.00	33.00	0.51	0,62
Age 1st sex cont	25	17.52	2.85	22	18.32	3.23	-0.89	0.38
Use condom at 1st sexual contact	25	0.52	0.51	22	0.91	0.29	-3.25	0.01*
At interview sex relation with partner	16	0.13	0.34	20	0.00	0.00	1.46	0.16
At interview sex relation with other than partner	26	0.77	0.43	22	0.91	0.29	-1.33	0.19
Number sex partner next to own partner	6	2.17	1.60	2	2.00	0.00	0.26	0.81
Does HIV prevention	26	0.19	0.40	22	0.32	0.48	-0.98	0.33
Way of HIV prevention	26	1.38	1.02	22	1.36	1.05	0.07	0.95
Partner tesedt for HIV	16	0.50	0.52	20	0.35	0.49	0.89	0.38
Result partner	16	0.88	1.15	20	0.85	0.88	0.07	0.94
Other sex partn tested	6	0.33	0.82	2	1.50	2.12	-0.76	0.58
Result other sex partn	6	0.17	0.41	2	2.50	3.54	-0.93	0.52
Talks with partner about HIV/AIDS	16	0.06	0.25	20	0.20	0.41	-1.24	0.23
When HIV pos tells partner	16	0.19	0.54	20	0.25	0.64	-0.32	0.75
Reaction when partner HIV pos and tells	16	1.06	0.85	20	0.65	0.75	1.52	0.14
Talks with children about HIV/AIDS or do so later	11	0.18	0.60	20	0.30	0.66	-0.51	0.62
Dichotoom education	26	1.54	0.51	22	1.45	0.51	0.57	0.57

* p < 0,01

In Table 3 correlations of the total group and the 2 age groups are given, comparing HIV/AIDS knowledge with different parameters. In group II there are significant positive correlations for better education, ways of prevention, and negative correlations for talking with offspring about HIV/AIDS, using tools for prevention, and reaction on HIV/AIDS positivity of partner. In group I a positive correlation is found for HIV/AIDS knowledge at 1^st^ sexual contact. There is a positive correlation between HIV/AIDS knowledge and years of education. But there is a negative correlation between a good HIV/AIDS knowledge and being tested for HIV/AIDS or using HIV prevention. Although farmers seem to have less knowledge about AIDS/HIV their use of condoms or other preventive measures is not less.

**Table 3:** Pearson product moment correlations between the total group and the 2 age groups

		HIV/AIDS knowledge			
			All ages	≤ 29 year	> 29 year
			Pearson Sig. (2-tailed)	Pearson Sig. (2-tailed)	Pearson Sig. (2-tailed)
**Age**			-0.183	0.078	-0.391
**Dichotoom**	Education		0.417**	0.313	0.530*
**Tested for**	Hiv/aids		-0.115	0.145	-0.403
**Age**	1st sexual contact		-0.068	0.293	-0.397
**HIV/AIDS knowledge**	1st sexual contact		0.311*	0.502*	a
**Number sex**	Partners		0.443	0.564	a.b
**Ways**	Prevention		0.355*	0.247	0.472*
**Partner**	Hiv/aids	Tested	-0.167	0.119	-0.394
**Condom**	1st sexual contact		0.148	0.275	0.000
**Religion**			0.015	-0.029	0.910
**Talks with**	Children	Hiv/aids	-0.404*	-0.194	-0.488*
**Does**	Hiv/aids	Prevention	-0.361*	-0.089	-0.614**
**Talks with**	Partners	Hiv/aids	-0.028	-0.092	-0.024
**Reaction**	Hiv/aids	Partner pos	-0.273	-0.027	-0.481*
**Job**			0.246	0.185	0.308

a: cannot be computed because at least one of variables is constant.

b: N = 2

* p < 0.05

** p < 0.01

There was no correlation with age, level of education, religion, or level of knowledge on HIV. There was no relation with testing on HIV of the 36 partners, of which 21 were tested (16 negative, 5 result unknown).

## Discussion

Our study showed that in a rural area in Cameroons South West province there is an overall good knowledge on HIV/AIDS and what should be done about it. Data are consistent with other studies. Remarkable seems that men with a high level of education and good knowledge on HIV/AIDS seem to do less to take preventive measures. Since better education is related to better welfare this is in harmony with other studies showing that HIV/AIDS is more frequent in wealthy men [10, 11, 13].

HIV/AIDS was first discovered in the early 1980ies [18–20] and general knowledge appeared years later. People becoming sexually active before that could not have known about HIV/AIDS. Therefore we compared men younger and older than 29 years of age separately, showing that age influenced the data on knowledge and behaviour.

Our demographic data are comparable to those in other studies, although the age group below 30 seems larger compared to those studies [5, 10, 15]. That group I had significant more knowledge about HIV/AIDS at the time of their 1^st^ sexual contact seems logic given the fact that HIV/AIDS was detected in the early 1980ies when at the time of the interview (2006) group II could not have heard about HIV/AIDS at the time of their 1^st^ sexual contact.

A survey among married men or those having a relationship showed that cohabitation is a common feature in Cameroon and that extra marital sex increased with age’> when at the time of the interview (2006) group II could not have heard about HIV/AIDS at the time of their 1^st^ sexual contact.

A survey among married men or those having a relationship showed that cohabitation is a common feature in Cameroon and that extra marital sex increased with age, education, wealth, urban versus rural and belief (Christian more than Muslim, but the latter are allowed to marry more women [10, 11]. These data also show that in contrast to South Africa in Cameroon HIV is more prevalent in the higher income groups and the better educated [10, 11, 13]. It also shows that there is a positive correlation between higher income and better education with earlier sex and unprotected extramarital sex. Of course there are considerable differences between these countries [13].

In Cameroon, as Awuba and Macassa showed, women are at increased risk of being infected with HIV/AIDS compared to men and that apart from biological vulnerability, socio-cultural as well as economic factors accounted for those differences [3]. In addition, they found that at the policy level, the government has drawn up plans to reduce the high prevalence of HIV/AIDS among women. However, although the current policy acknowledged the need for tackling gender differentials in HIV/AIDS transmission, little has been done at the level of implementation [3]. The current policy needs to be implemented in a more effective manner and a multisectorial approach should be explored in order to curb the current trend of the feminization of HIV/AIDS in Cameroon [3].

Muko et al demonstrated among HIV patients in Cameroon that paying for AIDS therapy is influenced by financial constraints [5]. Apart from cost, stigma, disbelief and side-effects of medication were found to be the main factors militating against willingness to pay [5].

De Loenzien demonstrated that in countries like Cameroon where confrontation with HIV is less, knowledge about it comes from information by indirect sources and that therefore men are better informed than women, because women tend to be less well-exposed to AIDS information campaigns than men [12]. She also showed that moving improved the knowledge about HIV/AIDS [15]. Recently, it was shown that both the integration of the family environment as well as the improvement of teach-the-trainer educational programs are essential for a better transmission of knowledge on HIV and AIDS [21, 22].

Limitations of our study are the small number of interviewed men of different background in a rural area, and the single observer who performed the interviews. Also, since the subject might be difficult for many to be confronted with, social acceptable answers might be given. Therefore our data are not representative for the whole country.

## Conclusion

In conclusion are our data on men in a rural area in Cameroon consistent with other studies in Cameroon on HIV/AIDS knowledge in men. Remarkable is the difference in HIV/AIDS knowledge between the 2 age groups, and the relation between HIV/AIDS knowledge and sexual habits and prevention. Sufficient HIV/AIDS knowledge did not lead to significant changes in sexual behaviour. The questionnaire used showed to provide sufficient information and it was easy to use. Further research on whether information or confrontation is the better strategy to inform people about HIV/AIDS and to change their behaviour should be performed.

## Additional material

**Annex 1:** Questionnaire items
